# A framework for the study of zoonotic disease emergence and its drivers: spillover of bat pathogens as a case study

**DOI:** 10.1098/rstb.2012.0228

**Published:** 2012-10-19

**Authors:** James L. N. Wood, Melissa Leach, Linda Waldman, Hayley MacGregor, Anthony R. Fooks, Kate E. Jones, Olivier Restif, Dina Dechmann, David T. S. Hayman, Kate S. Baker, Alison J. Peel, Alexandra O. Kamins, Jakob Fahr, Yaa Ntiamoa-Baidu, Richard Suu-Ire, Robert F. Breiman, Jonathan H. Epstein, Hume E. Field, Andrew A. Cunningham

**Affiliations:** 1 Disease Dynamics Unit, University of Cambridge, Madingley Road, Cambridge CB3 OES, UK; 2 STEPS Centre, Institute for Development Studies, University of Sussex, Brighton BN1 9RE, UK; 3 Animal Health and Veterinary Laboratories Agency, New Haw, Weybridge, Surrey KT15 3NB, UK; 4 Institute of Zoology, Zoological Society of London, Regent's Park, London NW1 4RY, UK; 5 Max Planck Institute for Ornithology, Vogelwarte Radolfzell, Schlossallee 2, 78315 Radolfzell, Germany; 6 Department of Biology, Colorado State University, Fort Collins, CO 80523, USA; 7 Division of Evolutionary Biology, Zoological Institute, University ofBraunschweig, Braunschweig, Germany; 8 Centre for African Wetlands, University of Ghana, PO Box LG 67, Legon, Accra, Ghana; 9 Wildlife Division of the Forestry Commission, Accra, Ghana; 10 US Centers for Disease Control and Prevention–Kenya Office, Nairobi, Kenya; 11 EcoHealth Alliance, New York, NY, USA; 12 Department of Animal Biology and Conservation Science, University of Ghana, Legon, Accra, Ghana; 13 Queensland Centre for Emerging Infectious Diseases, Biosecurity Queensland, Department of Agriculture, Fisheries & Forestry, Health and Food Science Precinct, Block 10, 39 Kessels Road, Coopers Plains, Queensland 4108, PO Box 156, Archerfield, Australia

**Keywords:** bat, zoonosis, emergence, collaborative framework

## Abstract

Many serious emerging zoonotic infections have recently arisen from bats, including Ebola, Marburg, SARS-coronavirus, Hendra, Nipah, and a number of rabies and rabies-related viruses, consistent with the overall observation that wildlife are an important source of emerging zoonoses for the human population. Mechanisms underlying the recognized association between ecosystem health and human health remain poorly understood and responding appropriately to the ecological, social and economic conditions that facilitate disease emergence and transmission represents a substantial societal challenge. In the context of disease emergence from wildlife, wildlife and habitat should be conserved, which in turn will preserve vital ecosystem structure and function, which has broader implications for human wellbeing and environmental sustainability, while simultaneously minimizing the spillover of pathogens from wild animals into human beings. In this review, we propose a novel framework for the holistic and interdisciplinary investigation of zoonotic disease emergence and its drivers, using the spillover of bat pathogens as a case study. This study has been developed to gain a detailed interdisciplinary understanding, and it combines cutting-edge perspectives from both natural and social sciences, linked to policy impacts on public health, land use and conservation.

## 1. Introduction

There is a growing awareness of the increasing threats presented to humans by emerging infectious diseases (EIDs) [1–3], with the majority of human EIDs being zoonotic—originating especially from wildlife reservoirs [4]. Emerging diseases have a huge impact on human societies across the world, affecting both current and future generations. Changes in human living patterns, along with environmental and climate changes, pose unprecedented challenges to the global health of people, animals and ecosystems. Ecosystem health correlates with human health [5], but the precise relationships remain poorly understood [6]. Understanding and responding to the ecological, social and economic conditions facilitating disease emergence and transmission represent one of the major challenges for humankind today [7]. The risk is not uniform [2]: 53 per cent of global EID outbreaks from 1996 to 2009 were in Africa, yet the continent lags behind severely in infectious disease detection and emerging epidemic warnings [8].

With increasing encroachment of people and livestock into wildlife habitats, a growing movement of wildlife from environmentally degraded areas into urban and peri-urban regions, massive aggregations of people (some at increased risk for severe infectious diseases because of AIDS, malnutrition, malaria and a variety of chronic infections) moving into densely populated cities, and rapid global movement of humans, animals and their products, there is a justifiable concern about the emergence and spread of novel, highly infectious diseases. Some of the most threatening emerging pathogens are RNA viruses due to their unparalleled ability to adapt to new hosts and environments [9]. Many RNA viral EIDs, including HIV-1, have emerged from wildlife, and an important implication of this is that the most effective place to address such zoonotic threats is at the wildlife–human interface. A key challenge in doing this is to simultaneously protect wildlife and their habitats, thereby preserving vital ecosystem structures and functions that have local and broader implications for human wellbeing and environmental sustainability, and to prevent the spillover of pathogens from wild animals into human beings.

In this multifaceted context, bats offer a critically important focus for study at the human–wildlife interface. Bats are an important reservoir and vector for spread of EIDs. Bats perform major ecological functions by pollinating plants and dispersing their seeds, as well as regulating insect populations that are critical for maintaining ecosystems; some have been recognized as ‘keystone species’ [10]. Yet bats are associated with zoonoses of potentially great global public health impact and are the source of lyssa viruses [11], Hendra virus [12], Nipah virus [13], severe acute respiratory syndrome (SARS)-like coronaviruses [14,15], and Ebola and Marburg viruses [16–19]; all are RNA viruses that can cause currently untreatable diseases in people, often with high case fatality rates. Bats frequently live in very close proximity to humans, often in large numbers. They often interact closely with livestock and other domestic animals that are potential intermediate hosts for viruses that can infect humans, thus effectively expanding the wildlife–human interface. These interactions are shaped by environmental, social and politico-economic drivers at multiple scales, yet these processes and interrelationships are poorly characterized and understood. Bats epitomize growing challenges associated with human–wildlife–disease interactions, and thus offer a valuable model for building a new, holistic, policy-engaged paradigm to address these, now and in the future.

A clear institutional framework has recently been proposed for responses to emerging zoonotic diseases that require a multidisciplinary, ‘one health’ approach for their management [20]. Such an approach recognizes the interdependence of human health, animals and ecosystems, but provides little guidance for researching these in an integrated manner. More specific inclusion of the drivers of spillover events is essential if we are ever to use our research to develop long-term programmes and strategies that reduce the future likelihood or frequency of spillover events. Further, approaches for the study of these complex ecological events must include study of the relevant institutions themselves, as their policies shape local and larger scale responses and perceptions.

Vitally needed for the full, long-term addressing of the risks of bat (and other wildlife) derived zoonoses is therefore an approach that gains detailed interdisciplinary understanding, combining cutting-edge perspectives from both natural and social sciences, linked to policy impacts on public health, land use and conservation. There needs to be greater support for new approaches that cross disciplines and combine quantitative and qualitative methods, and that also directly address the politics of policy processes. Such an integrated approach will be critical to future efforts that address disease challenges at the human–wildlife interface. Here, we propose such a framework, using bat-related disease threats as an example.

## 2. Why bats and humans? what are the challenges?

RNA viruses associated with Old World fruit bats pose zoonotic disease threats of high public health significance internationally. We propose that pathogen spillover occurs from bats to humans and affects public health, but the dynamics, effects and extent to which spillover is recognized, and responded to, depend on varied combinations of biological, environmental, social and politico-economic processes and drivers.

New metagenomic studies of viral abundance and diversity in bats [21,22] (Baker *et al*. 2012, unpublished data), as in other species [23–25], have demonstrated the amazing breadth and diversity of microbial populations in different bat species. An unknown proportion of the detected infections will have the ability to cross the species barrier, with or without adaptation [26]. The spillover infection dynamics will, however, be very different between different species of bat and microbe, with additional and marked geographical variation influenced by environmental factors and human behaviours.

Existing knowledge about spillover infection dynamics is generally very patchy. While many studies have been sufficient to establish the public health importance of the spillovers in a local context, many important puzzles remain, particularly when considering larger scales [27]. For example, bat lyssaviruses have been associated with fatal human encephalitis on almost every continent, while outbreaks or cases of encephalitis caused by henipaviruses have been confirmed in Australia, Southeast Asia and South Asia. Until recently, this distribution of outbreaks was expected as henipaviruses were thought to be confined to Australasia, southern Asia and Madagascar [28]. However, the discovery of henipaviruses in Africa [29,30] greatly increases the geographical range for the potential spillover to humans and other animals, adding to the recognized threats of lyssaviruses and filoviruses from bats in Africa [16,19,31–35]. Indeed, spillover in Africa may already be occurring; encephalitis often goes undiagnosed and defining its causality is not straightforward [36,37]. Studies increasingly demonstrate that cerebral malaria is often over-diagnosed and rabies under-diagnosed [38], as henipavirus or other unknown viral diseases might be. As recent reports of widespread seroprevalence to Ebola Zaire in healthy villagers in Gabon underline [39,40], some preconceived notions regarding the diagnosis, surveillance and potential for emergence of ‘feared’ pathogens are open to challenge. Knowledge of the infection dynamics of these pathogens in their natural hosts is essential to increase our understanding of spillover dynamics and to assess fully the implications for, and the protection of, public health.

Zoonosis-related health issues also highlight deeply understudied social and environmental questions. The presence of fruit bats in cities in Africa, Asia and Australia, for instance, demonstrates their ability to adapt to changing environments, but also increases human–bat interactions, adding to those occurring through rural land use, livelihoods and occupations. But there is little understanding of the social, economic, political or environmental dynamics and drivers at different scales that shape these interactions, or of the beliefs, understandings and cultural practices which surround human–bat contact. How local people and national and international policy-makers perceive bats and associated disease risks, and how these different ‘framings’ might mould or impact public health policies, is very unclear. Framing refers to the ways in which scientific topics and policy processes are delineated. Cultural contingencies, life experiences, intellectual paradigms and political agendas are often highly influential in shaping how science or policy is conceptualized. These unrecognized ‘blinkers’ limit the possibilities to recognize multiple perspectives, seek more participatory solutions or question the assumptions on which decisions get made. As Jasanoff [41] points out, cognitive frames ‘impose discipline on unruly events by creating understandable causal relationships, identifying agents of harmful behaviour, and finding solutions that convey a sense of security and moral order’. Leach & Scoones [42] thus argue for the necessity of recognizing other kinds of knowledge shaped, or ‘framed’, through ‘other practical cultural assumptions, meanings and life-worlds’. Understanding how, why, where and for whom bat zoonoses pose problems, particularly in regions where public health surveillance is patchy or under-resourced, and how policies and interventions should address these, necessarily requires an integrated, interdisciplinary conceptual framework, informing specific questions to be investigated across a diversity of global settings.

## 3. Why an integrated approach?

A new holistic paradigm integrating biological, social and environmental science approaches is required to explain the mechanisms and impacts of zoonotic emergence, particularly through intermediate hosts. Novel mathematical frameworks have highlighted how chance events in viral evolution and transmission can lead to successful spillover [26,43]. Ecological models for zoonotic emergence are still very patchy [26,27] and need further development. Together, mathematical ecology and epidemiology provide mechanistic frameworks to study the dynamics of infections in bat reservoir populations [44]. Social science perspectives, especially from anthropology, are needed to elucidate how people perceive and interact with bats. Environmental science and modelling are necessary for addressing the ecological drivers of change that may impact on bat populations and hence spillover risk. Public health, social science and science–policy perspectives are important to consider how these diseases may be diagnosed, or continue undetected in humans, how policies and responses are framed and the political–economic interests that might influence this. This combination of disciplines needs to be carefully integrated in a manner that has not really been achieved for the study of any disease, let alone a wildlife-associated zoonosis. Here, the central involvement of bat conservation organizations could also enable synergies between science and policy and provide clear pathways to impact.

In policy terms, ‘one health’ approaches rest on the shared principle that the health of humans, animals and ecosystems are interdependent. However, in practice, there is little integration in addressing these different dimensions, while policy responses continue to reflect sectoral divisions. There is a clear need to integrate interdisciplinary research and policy, and to work across wildlife, veterinary and public health sectors, to develop approaches and interventions geared to enabling people and bats to coexist with a reduced disease risk to humans and livestock. There is also a need for evidence-based, practical techniques and public health interventions to mitigate the threats posed by bat viruses, while improving knowledge and understanding of the importance of fruit bats for ecosystem function and sustainability. A proposed framework for how such an integrated research–policy approach might be developed is laid out in the following sections.

## 4. Systems framework

**Figure 1.**
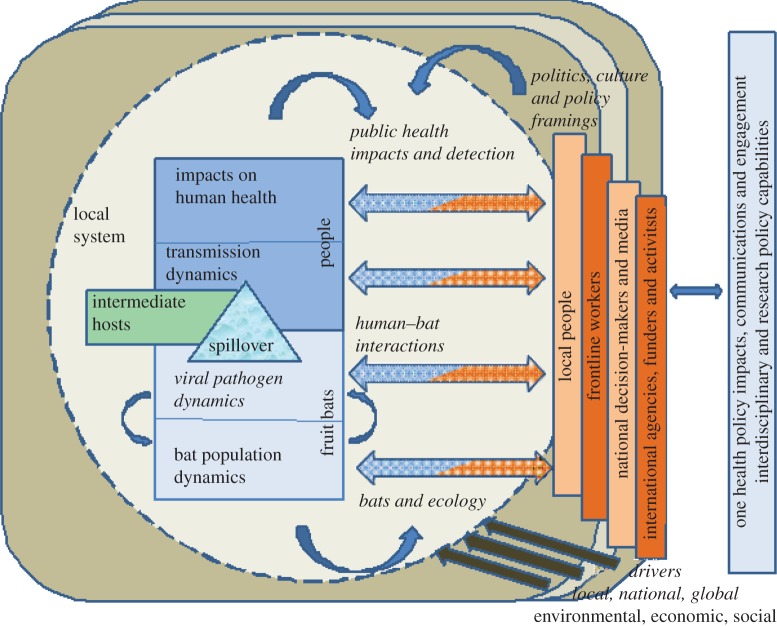
A conceptual framework for the study of wildlife derived zoonoses, focused on bat infections.

Work needs to be organized from the outset in a manner that both recognizes and respects individual disciplinary approaches, but that also cuts across them in a truly interdisciplinary manner in order to deliver genuine integration, both between disciplines and across localities. Although frequently ignored in the natural sciences, we propose a clear interdisciplinary conceptual framework (as is more common in interdisciplinary social science work, such as development studies) to capture the integration across all scales that is required (figure 1). Our framework integrates dynamic interactions between bats, viruses, intermediate livestock hosts and people in a local system, influenced by wider environmental, social and politico-economic drivers. Figure 1 is inspired by a range of research fields, drawing together perspectives from medical and veterinary (virology, epidemiology, public health), environmental (ecology, biodiversity) and social (anthropology, politics, science–policy studies) sciences. Each key element of the framework comprises a potential research theme within which specific questions can be investigated; importantly, novel insights and policy impacts should be derived from their integration.

Thus, in figure 1, the central rectangle portrays the inter-linked spectrum of dynamics involved in zoonotic spillover and disease emergence: bat population dynamics, their effects on viral pathogen dynamics, the dynamics of human exposure [45–47], including the involvement of domesticated species and the effects of the pathogens on human health and wellbeing.

The dynamics of individual spillover events are based around the concept of the pyramid (triangle here) of pathogen emergence first proposed by Antia [26] and developed further by others, including Lloyd-Smith *et al.* [27]. It is proposed that humans are constantly challenged by animal-derived pathogens (the so-called pathogen, or viral, ‘chatter’), but that only a small proportion manage to invade individual humans. Of those invading, most will be controlled by innate immunity and not replicate efficiently. Of those (proportionally) few that manage to replicate within the new host, most will not be able to transmit between individuals, or will do so only very poorly. As reviewed [27,48], the major determinant of whether these replicating pathogens will then invade human populations is the rate at which they can transmit between humans.

Our framework also captures how intermediate hosts, such as livestock, can play key bridging roles in such spillover events. Bridging species have been particularly important for many of the most serious bat-derived human pathogens, including the horse for Hendra virus [12], the pig for the initial emergence of Nipah virus [49], non-human primates in the case of Ebola [50,51] and the palm civet in the case of SARS-coronavirus [52]. The disease impact on bridging species can have severe human wellbeing implications in its own right, as exemplified by the decimation of the pig industry in Malaysia as a consequence of Nipah virus emergence [53].

Spillover dynamics are subject to a range of local influences and practices, both social and environmental, including *environmental influences*^1^ on viral pathogen dynamics, such as interactions with susceptible sympatric species. *Land use, wildlife management and conservation practices* can shape bat ecology and populations. The interactions between bats and ecosystems are manifold; through seed dispersal and pollination, bat populations also influence ecological structure and functioning. Infection dynamics are shaped by (and can, in turn, feed back to shape) *bat ecology* and related ecosystem processes; in turn, infection dynamics influence spillover dynamics. *Human–bat interactions*, including livelihood and ritual practices, bring different people into contact with bats and potentially expose them to disease. The public perception of bats and bat diseases can trigger eradication efforts that may then increase spillover risks. *Public health impacts and detection*, including disease surveillance and diagnostics for known pathogens (and capacity to detect previously unrecognized pathogens through newly evolving ‘pathogen discovery’ techniques [21,22]) and health-seeking practices, shape whether human infections with bat-derived pathogens are recognized. Such local system dynamics are shaped by wider *drivers* of change (environmental, social, political and economic), operating across different geographical scales. Importantly, our framework integrates a focus on *political, cultural and policy framings*, examining how different people in communities and in national and international agencies understand and represent spillover dynamics, public health threats and influences, and how these framings shape policy responses. Finally, we attend to how local system dynamics are shaped by wider drivers of change—environmental, economic, demographic, social—operating across local, regional, national and global scales. Taken together, bats provide a model for these framework elements, which should provide the evidence required to inform a series of ‘one health’ interventions and policy impacts, and assist the building of new interdisciplinary capabilities for research, policy engagement and disease mitigation while also enabling the conservation of biodiversity.

## 5. Research themes

There are a number of ways that specific research themes could be developed within our proposed conceptual framework. A thematic approach is critically important as it allows intra-disciplinary study, vital for ensuring impactful and relevant publications, each testing specific hypotheses. Such outputs are the building blocks for substantial interdisciplinary programmes and, while not every output will incorporate obvious interdisciplinary approaches, each should be informed by them. The same themes can cross geographical divides, providing integration on that scale. All these factors are critical for scientific inference as well as for evidence-based international and national policy development.

Here, for the specific example of the study of Old World fruit bat-related zoonoses, we propose six specific research themes by way of illustration. Each of the six themes is discussed below in more detail, highlighting key conceptual foundations and literatures, with some questions that could be addressed and the core, generic methodologies required.

### (a) Bats and ecology

This theme encompasses the framework elements that focus on Old World fruit bat ecology and population dynamics, and their interactions with ecological structure and function.

Critical questions include:

— What are the distributions, abundances, behaviours and feeding ecologies of the focal bat species?
— How do anthropogenic impacts, such as habitat alteration, urbanization and hunting, affect the distribution, abundance, behaviour and feeding ecology of focal fruit bat species?
— How do life histories, including quantitative population dynamics, and feeding behaviours of focal bat species influence the potential for viral spillover into human and domestic animal populations?

Bat species differ markedly in their ecologies, which may influence spillover. For example, in sub-Saharan Africa, *Eidolon helvum* and *Rousettus aegyptiacus* are the most widespread and possibly the most abundant fruit bats, often living in colonies of up to several million individuals. *E. helvum* often roosts in trees in urban settings, whereas *R. aegyptiacus* roosts predominantly in caves and in more rural areas [54,55]. *E. helvum* is migratory, probably following the burst of fruits and flowers with the onset of the wet season [56], but where they go during this time is largely unknown [57]. While some individuals have been shown to migrate over 2500 km [58], not all individuals migrate. In West Africa, *E. helvum* colonies can be very large (with roosts of more than one million individuals), while in East Africa *E. helvum* colonies appear to be smaller and more fragmented with reportedly less-pronounced migratory behaviour. In Southeast Asia, pteropodid fruit bats also may be highly mobile, though are sometimes perceived to be sedentary, living in small, fragmented colonies [59–61]. In Australia, flying foxes often live in very large, shifting colonies [47]. All these species have one pup per year during a synchronized birth pulse. In Bangladesh, where Nipah virus spillover occurs annually [62,63], and in Asia, there is a temporal association between bat reproduction and potential zoonotic spillover events [47,64]. In West Africa, *E. helvum* bats probably birth and mate during migration [65–67], which might be linked to, or even driven by, the nutritional needs of the females and their offspring, but the timing and place of these remain largely unknown [65,68]. It is possible, therefore, that spillover events occur on the migratory route of this species. Knowledge about migration, time and place of the reproductive cycle in conjunction with the number of animals at any given time and place, and resource availability will provide crucial information about these ecological keystone species, and point to where and when potential spillover to humans should be researched. Alternatively, bats may use migration to escape from areas with high disease load, or lower pathogen prevalence during migration [69].

The ecology and distributions of fruit bats in many countries in which spillover may occur are not very well characterized, particularly quantitatively. This is the case even in Australia, although huge advances have been made there in recent years [47]. The study of zoonotic pathogens has stimulated the study of a number of species, including *Pteropus giganteus* [70] and *Pteropus vampyrus* [71] in India, Malaysia and Bangladesh and *E. helvum* in Ghana [72,73]. In Australia, however, where Hendra virus spillover could come from any of four fruit bat species, the role of sympatry and cross-species virus transmission in driving spillover has not been elucidated at all [46]. A first necessary focus in many regions is the development of national schemes to locate, count and monitor bat colonies of focal bat species, to determine migratory patterns and to assess the reproductive cycle and efficiency. Quantification of social interactions between bats (e.g. mother–offspring, mating, fighting, allogrooming, etc.) would provide information on possible virus transmission routes. A second essential focus should be to understand feeding behaviour and ecology, as undertaken already in Bangladesh [74]. The use of novel high-resolution GPS data loggers allows detailed and quantitative studies of ranging behaviours of bats and their environmental determinants. Such methods would also underpin the identification of food plants and allow resource use to be quantified through faecal analyses [75]. The importance of fruit bats to the structures and functions of local ecosystems is often very poorly characterized; improving our understanding of this will inform how bats influence ecosystems and how land use change might influence bat population—and consequently infection—dynamics (see below).

The lack of longitudinal population data for most bat populations limits our understanding of the impacts of anthropogenic change on the ecology and behaviours of bats, but comparisons of single species living in both urban and rural sites, particularly where there are variable exposures to different degrees of hunting pressures, can help to evaluate these. Studies tracking movement patterns [58,59] (Dechmann and Fahr, unpublished data) can enable detection of temporary stopover roosts and allow resource availability to be linked to movement, reproduction and local bat population size. Importantly, identification of feeding sites can facilitate the determination of interactions with other wildlife species (especially other species of bat) and with livestock and humans (directly and through partially eaten fruit and fruit spats).

### (b) Viral pathogen dynamics

Determining the processes by which viral pathogens are transmitted within bat populations, and spillover between bats and other animals, is a critical step towards understanding spillover to humans and its regional and temporal variation. Critical questions include:

— Is there evidence for endemic circulation of zoonotic viruses in bat populations and how is such circulation affected by bat population dynamics and life history?
— What aspects of viral pathogen dynamics in bats and bat–domestic animal interactions influence the likelihood and frequency of zoonotic spillover events?
— What environmental factors drive viral pathogen dynamics and spillover between bat species and across locations and seasons?
— Do bats harbour an unusually wide range of viruses highly pathogenic in other species?

The development of mathematical models that integrate information from all the empirical research components is a critical foundation of this theme [44,45,47,49]. These models should investigate the potential roles of different mechanisms that can influence infection dynamics, including host–pathogen interactions at the individual level, virus circulation within and among bat colonies (which requires detailed understanding of host ecology) and inter-species transmission (impacted by viral tropisms and human–bat interactions).

Beyond their traditional use for *post hoc* analysis of data, mathematical models are critical, primary elements in this research framework on disease spillover that can provide:

— rational guidance for field data collection, particularly for wildlife disease [44]. This can maximize the utility of quantitative information and its use in models; and
— a quantitative framework to connect the different research themes and disciplines and to analyse their impact on virus dynamics.

Pathogen dynamics must be surveyed longitudinally in bat populations, using the appropriate (for the pathogen) suite of serological and virological techniques. The importance of longitudinal data from carefully selected bat populations at sufficiently frequent intervals, preferably including some measures of age-specific infection rates or seroprevalence, cannot be overemphasized. These investigations should be conducted in parallel with serological, virological and epidemiological studies in relevant human and domestic animal populations to determine the occurrence of spillover.

Quantitative virological and serological approaches are needed to provide data for the parameterization of mathematical models [45,46]. Particular challenges of this type of work include the collection, storage and transportation of samples in a manner suitable to allow subsequent testing and the very high biocontainment levels needed to work with samples where BSL3 (e.g. lyssaviruses and SARS-like coronavirus) and BSL4 (e.g. filo-, and henipa-viruses) pathogens are present.

Much of this work should be massively enhanced when the promises of cheap, quantitative and highly sensitive chip-based pathogen detection systems [76] become validated in multiple species to allow parallel studies in bats and sympatric species including humans.

### (c) Human–bat interactions

Human–bat interactions are critical to shaping people's exposure to zoonotic pathogens, but this key driver of spillover has been insufficiently studied. Relevant questions include:

— How do people perceive and interact with bats, including through intermediate hosts and indirect contact with bat secretions, according to their living environment and via livelihoods, bushmeat consumption and ritual practices?
— How do these practices differ by gender, ethnicity, age and wealth, and so shape differential risks of disease exposure?

Such thematic research should most obviously be grounded in ecological anthropology, political ecology [77,78] and livelihood approaches [79], seeking to understand human–bat interactions in their ‘biocultural–political complexity’ [80]. Although there is a growing body of livelihood-focused literature (e.g. on bats and bushmeat [81,82]), specific mention of bats is very limited in general bushmeat studies [81,83]. Early ethnographic accounts focused on material culture, such as the construction of nets for bat-catching [84] and on structural analyses of people's classification schemes of fauna and flora [85–87]. Anthropological research on foraging and hunting notes the presence of bats as a source of food [88], and frequent reference is made to bats' ritual, symbolic and liminal roles [89–92], but in-depth research seems limited to the examination of bats' medicinal powers in Bangladesh [93] and to the exploration of ecosystem interrelationships between monks, bats and caves in Thailand [94].

Research should critically explore people's perceptions and practices in interacting with bats [95], perhaps drawing inspiration from recent anthropological theorizing on the social character of human–animal interactions [96–99], considering livelihood trajectories [100], gendered strategies [101] and differential access to resources and decision-making.

Understanding material interactions with bats, the presence of bats within local belief systems and folklore, attitudes and responses to the existence of bats near dwellings and domestic animals, and the sharing of environmental resources with bats could shed particular insights into zoonotic disease mitigation when considering future policy development. Geographical and temporal overlap of such sociological research with parallel ecological studies (outlined earlier) would be particularly valuable.

### (d) Public health impacts and detection

The practices and health system factors that influence how prevalent spillover risks are, and how these are detected in different settings, need specific study in most developing world situations. Key questions here include:

— To what extent do Old World fruit bats present EID threats to public health?
— What epidemiological links exist between human demographic and behavioural factors (including livestock interactions) and spillover?
— How are disease detection and diagnosis shaped by surveillance, health system and health-seeking infrastructures and practices?

In this challenging theme, the obvious epidemiological and public health approaches must be integrated with anthropological perspectives that emphasize how prevailing social and cultural values, legal, political and economic factors and organizational norms influence disease classifications and diagnoses [102,103]. Critical medical anthropology [104] has directed the attention towards the social and political determinants of ill health, disease distribution and access to health care. Recent approaches in health systems research understand health care systems not merely as structures of services, goods and personnel, but as knowledge economies [105] involving health markets that include formal and informal practitioners, with a range of factors influencing people's understandings of illness and health-seeking behaviour. These concepts should underpin the investigation of the extent to which bat virus spillover events are recognized by a range of groups and how diagnosis and public health responses are shaped by institutional factors and health care workers' practices. From socio-epidemiological perspectives, an investigation of how behavioural and socio-demographic factors can predispose people and their domestic animals to spillover infections would be very valuable. Relevant biomedical syndromes must be considered alongside enquiry regarding the extent to which zoonotic disease risk is recognized by local people and features in their concepts of illnesses and perceptions of relevant causes of morbidity and mortality.

The detailed laboratory investigation and confirmation of specific infections in humans must follow the same detailed criteria as used for defining the specific infections in bat hosts. Careful quantitative planning and consideration of control selection will be vital for the successful interpretation of data from human patients in epidemiological studies.

If understandings of symptoms caused by infections from bats exist, or when epidemiological links with bats in clinical cases are found, possible routes of transfer can then be investigated—and triangulated with the studies of *human–bat interactions* described earlier. People's attitudes to past disease surveillance measures and health education initiatives should be elicited to assess the extent to which these might influence contemporary illness concepts and attitudes. Beliefs about bat-associated diseases on the part of health care workers need to be recorded alongside observation of clinic culture and diagnostic practices. The importance of this was exemplified in Bangladesh, where bats were rejected as a source of Nipah virus in favour of superstitious causes, even among health-care workers, which obviously then can impede containment and control measures [93].

This thematic research area can raise particular methodological and potential ethical issues, as people living in close interaction with bats may not realize the potential for spillover. The immediate reaction to this knowledge is often to want to get rid of the bats, but there are interrelated livelihood challenges, value system and cultural challenges and ecological challenges that are not always initially recognized. Thus, context-specific sensitivity and careful sequencing of enquiries is necessary.

### (e) Drivers

*Drivers of spillover*, as shown in figure 1, may include environmental and climate changes, as well as socio-economic, demographic and political drivers of relevant processes, such as agricultural intensification, livestock keeping or bushmeat hunting [47,106,107]. Such drivers are rather poorly understood for most zoonotic systems, so even improved qualitative study would be valuable, asking:

— How are local pathogen spillover events, Old World fruit bat ecology and human–bat interactions influenced by wider drivers, operating at local, national and regional scales?

Contextual descriptions of key drivers identified for each locality should be constructed by drawing on existing environmental, social and historical literatures, including qualitative analyses, to track up from key processes identified in the *human–bat interactions* and *bats and ecology* themes to identify national and international influences on these. Optimally, this would be complemented by empirical modelling techniques [2,108] to investigate the correlation of disease spillover events and their drivers at different spatial scales. Spatially explicit ecosystem data, such as land use, wildlife densities, livestock densities, human population densities, climate and socio-economic variables are increasingly available for such analyses [2,108]. With a careful interpretation, empirical models can quantify the impact of different spatial drivers on risk of initial spillover and subsequent spread and at what spatial scales these may be important. On the basis of time series analyses, risk maps could be generated that predict spatio-temporally changing ‘hot zones’ for spillover, thus contributing to forecasting.

### (f) Politics, culture and policy framings

Building on more local focus in the *human–bat interactions* and *public health impacts* themes, work here should explore broader interpretations and representations—framings—held by a wide range of different interested parties (often termed ‘actors’ in the social sciences) across multiple scales:

— How do local, national and international actors frame bat–human–disease interactions, spillover and risks according to their cultural backgrounds, institutional positions and political interests?
— How do these framings shape responses, policies and interventions?

This work could be valuably informed by social science and science–policy perspectives on framing and by the construction of (policy) knowledge through social and political processes and cultural logics [41,109,110]. It has been shown that framings often take the form of ‘narratives’ or underlying storylines which drive and justify different kinds of intervention and response [111], including in relation to disease and epidemics [111,112]. Different framings are associated with different actors, institutions and policy communities, linked in different ways across scales [113]. Depending on the actors and the power relations between them, policy and intervention practices will differ, and therby shaping spillover dynamics and public health impacts.

The framings of interest here refer to bats, including their value and the threats they pose, human–bat interactions, public health impacts (incorporating both historical outbreaks and future threats) and the very notion of ‘spillover’ and how it occurs. Ideally, such framings should be investigated for a range of actors, including local people (differentiated by gender, age and occupation, such as bat hunters, ritual specialists and livestock keepers); front-line government and non-governmental organization (NGO) professionals and practitioners (in the public health, veterinary and wildlife sectors); national policymakers and media; and international agencies concerned with bats, environment and health (including the United Nation's World Health Organization (WHO) and Food and Agriculture Organization (FAO), the World Wide Fund for Nature and the International Union for Conservation of Nature (IUCN) Bat Specialist Group). Data from these studies need to be analysed systematically through qualitative techniques to identify key framing and narrative elements and clusters, to elucidate their relation to actors' cultural backgrounds and political–institutional positions, and to draw out key lines of contestation and their implications. This thematic work could be particularly influential for policy-related work that might follow, aimed at informing and inspiring shifts in existing framings and in new policy approaches.

## 6. Implementation and impacts

An integrated programme of this nature, especially if undertaken on Old World fruit bat infections, will usually be in the developing world, and involve locally based scientists. In order to achieve a truly integrated and interdisciplinary programme, the simple creation of teams and the development of cross-cutting research themes, as mentioned earlier, that focus on integrative processes is insufficient [114,115]. It is essential that dedicated capabilities among programme members are developed for interdisciplinary work and analyses, and this is particularly so for linking research with policy impacts. Building such capabilities within local institutions is a further key element of the conceptual framework proposed here (figure 1). An important aspiration should be to deliver capacity-building plans that create learning relationships among programme members and collaborators: (i) from different disciplinary and natural/social science backgrounds; (ii) from scientific and more policy-oriented backgrounds and institutional positions; and (iii) in both senior and more-junior positions. Of course, the challenge is to develop a relevant suite of effective activities to deliver this that exploits both tried and tested traditional approaches as well as innovative web-based type activities. Regular face-to-face interactions between team members, including joint fieldwork, will always remain a critical component of effective delivery plans.

Delivering ‘one health’ approaches and intervention options with the potential for significant impact on policy and practice should be a central process in any integrated interdisciplinary programme of research into zoonotic disease emergence. There will frequently be strong demand for such work among potential users and beneficiaries. In relation to bat infections, these might include communities, occupational groups (e.g. healers, bat hunters), conservation associations and ‘frontline’ health practitioners. Nationally, they would include government environment, wildlife, veterinary and public health departments, NGOs and industry groups (e.g. horse owners in Australia). Internationally, they would include organizations addressing human health (e.g. WHO), animal health (e.g. FAO, OIE), biodiversity (e.g. IUCN, IPBES, CBD, CMS, TEEB) and bats (e.g. Bat Conservation International, BatLife Europe, EUROBATS).

Other than creation of new knowledge, what might the desired impacts of a programme of this nature be? A number of areas should be addressed, including the development of practical techniques to enable local people to live safely with bats with reduced risk of disease transmission. Integrated, cross-sectoral national policy approaches should inform wildlife, environmental and public health policies. Better-informed public and media debate about bats and bat-related disease will markedly improve understanding in many groups and so facilitate communication. Broader ‘one health’ lessons and approaches will aid international science and policy communities, and methodologies for a new holistic paradigm could produce a lesson-learning manual for research teams and agencies engaged in interdisciplinary environment and health challenges. Specific strategies for research impact should be defined at local, national and international scales. These should identify and incorporate likely users of the research from the earliest stages in refining potential outputs, communications and uptake plans. Such inclusive planning can be facilitated by specific participatory exercises—for example, Participatory Impact Pathways Analysis (http://impactpathways.pbworks.com/w/page/19812765/FrontPage).

## 7. Conclusions

We describe a framework for the holistic study and management of the emergence of disease from wildlife, focusing on Old World fruit bats as a model. Inter-disciplinary approaches are vital, but do not remove the importance of more reductionist studies. The optimal approach will always depend on the precise questions being asked. For example, if the requirement is to undertake a risk assessment of the ability of a range of pathogens in one ecosystem to infect humans, then a study focused on understanding host–pathogen interactions, probably initially considering pathogen receptors, would be appropriate. Mechanistic understanding that might be derived from such studies may be important in assessing the ability of particular pathogens to spill over, but does not give insight into the ecology (e.g. types and degrees of exposure) or the rates that the target pathogens are able to cross species barriers.

Detailed collaboration between mathematics and natural science is now well established in the study of pathogen dynamics, and indeed is at the heart of our proposed framework. We propose that further integration of both disciplines with the social sciences can produce further benefits. Added value can come from working with other disciplinary approaches; for example, for the natural sciences, understanding of the social factors shaping the dynamics of interest, as well as more explicit and effective addressing of policy issues within the research framework are obvious benefits from working with social sciences; for the social sciences, more detailed understanding of the biological processes of interest can both raise vital new questions and beneficially refine research approaches. We suggest that holistic, integrated and interdisciplinary studies, as proposed here, could produce a step change in our understanding of how best to deal with the complex issues surrounding disease emergence, especially from wildlife.

## Endnote

1 Italicized text corresponds to elements in figure 1.
